# Cystic Echinococcosis: Chronic, Complex, and Still Neglected

**DOI:** 10.1371/journal.pntd.0001146

**Published:** 2011-07-26

**Authors:** Enrico Brunetti, Hector H. Garcia, Thomas Junghanss, on behalf of the members of the International CE Workshop in Lima, Peru, 2009

**Affiliations:** 1 Division of Infectious and Tropical Diseases, University of Pavia, IRCCS S.Matteo Hospital Foundation, WHO Collaborating Centre on Clinical Management of Cystic Echinococcosis, Pavia, Italy; 2 Cysticercosis Unit, Instituto Nacional de Ciencias Neurológicas, Lima, Peru; 3 Department of Microbiology, School of Sciences, and Center for Global Health, Universidad Peruana Cayetano Heredia, Lima, Peru; 4 Section Clinical Tropical Medicine, Department of Infectious Diseases, University Hospital, Heidelberg, Germany; New York Blood Center, United States of America

## The Overall Scene

**Figure 1:**
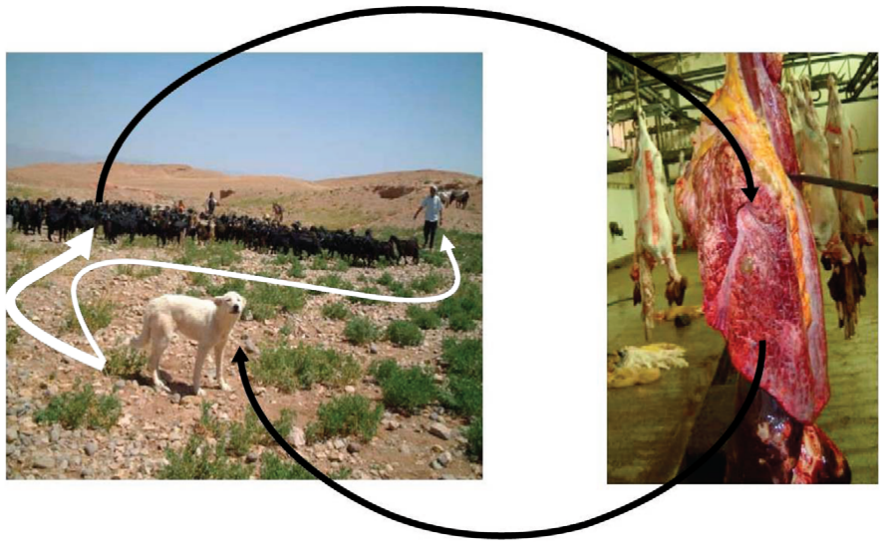
Life cycle of *Echinococcus granulosus* in a community of the Middle Atlas region, Morocco. (We thank M. Kachani, College of Veterinary Medicine, Western University of Health Sciences, for the pictures.)

Cystic echinococcosis (CE), an infection with the larval form of the dog tapeworm *Echinococcus granulosus*, still causes serious lung and liver disease with a worldwide geographical distribution. This parasitic infection is preventable, eliminable, and treatable—in theory. The biological cycle can be attacked at various points: regular dog deworming, controlled sheep slaughtering, vaccination of the intermediate (sheep) animal host, and possibly in the future, vaccination of the definitive (dog) animal host (Figure 1). However, breaking the cycle in practice is difficult and requires long-lasting efforts. Control programs are expensive to set up and sustain. With the currently available options, a period of 20 years is needed to reach elimination, a goal that, unsurprisingly, has only been reached in rich countries [1].

At the current pace of control, patients suffering from CE will be seen for many decades to come. CE disease is chronic, complex, and neglected [2]–[4]. It is still poorly understood, and recommendations for diagnosis and treatment have not progressed beyond expert opinions and are not necessarily adopted by clinicians because of lack of grade I evidence.

The critical issues are:

1. CE may develop silently over years and even decades until it surfaces with signs and symptoms or as a chance finding on an ultrasound (US) scan or chest X-rays requested for unrelated reasons. Clinical manifestations may mean that the cyst is already complicated, e.g., ruptured into the biliary or bronchial tree, secondarily infected with bacteria, or leaking and causing allergic reactions if not anaphylactic shock.

2. Screening large samples of populations to detect asymptomatic cases is expensive. As with all screening procedures, ethical issues arise: do all patients in whom cysts are found require treatment? Is the treatment which we then offer well established and safe? And is it available at all? Screening projects in endemic areas are often inadequately prepared, as the clinical management is not provided locally for those who are found positive.

Problems start with the screening tool. With the exception of liver US, the available methods are far from satisfactory. In regards to serology, the sensitivity and specificity of several antigens have been well defined [5], [6], but available assays still lack standardization, sensitivity, and specificity [7]. Controversies on the usefulness for clinical diagnosis and screening remain unresolved [8]. Serodiagnostic performance depends on several factors, such as cyst location, cyst stage, and even cyst size, but these and other variables have not been thoroughly assessed to date.

Ultrasound is an indispensable tool, but will likely miss very small cysts, and its efficacy is mostly restricted to intraabdominal organs. Additionally, some cyst stages may be difficult to distinguish from non-parasitic cysts, which are common. The problem continues when an echinococcal cyst has been diagnosed. In settings where health care facilities are several days of travel away from the rural areas where patients live and work, and as long as we have doubts on what the natural evolution of their cysts will be, clinical decision making is difficult. It has to be done in each case individually based on current standards, clinicians' experience, and local technical possibilities, supported by embarrassingly poor evidence.

3. Not all CE patients are similar, even at a population level. Broadly speaking, there are two defined groups of patients, each with a different set of problems: mainly asymptomatic patients (detected in screening programs or by chance), or clinically apparent cases (mostly patients with complicated cysts).

*(a) Patients with cysts detected during screening activities or as a chance finding.* They mostly receive the treatment with which the attending clinician is familiar. This is not necessarily the best option relative to the cyst stage and clinical situation of the patient. Preliminary results from a survey on knowledge, attitudes, and practices regarding clinical management of CE in European, North African, and Middle Eastern countries yielded alarming results [9]. Patients may be put at risk of interventions that may be completely unnecessary. This certainly applies to a sizeable number of cysts that have become inactive and do not cause any symptoms or complications.

A significant proportion of cysts stop growing and follow a path to spontaneous involution. Long-term follow-up suggests that these cysts and the patients harbouring them should be left alone. This is an appealing perspective for patients and health services, if evidence can be gathered in its support. CE4 and CE5 cysts appear to be very good candidates for this approach if they do not compromise any vital structures. It is, however, unclear if and under which circumstances this concept can be extended to other cyst types.

**Figure 2:**
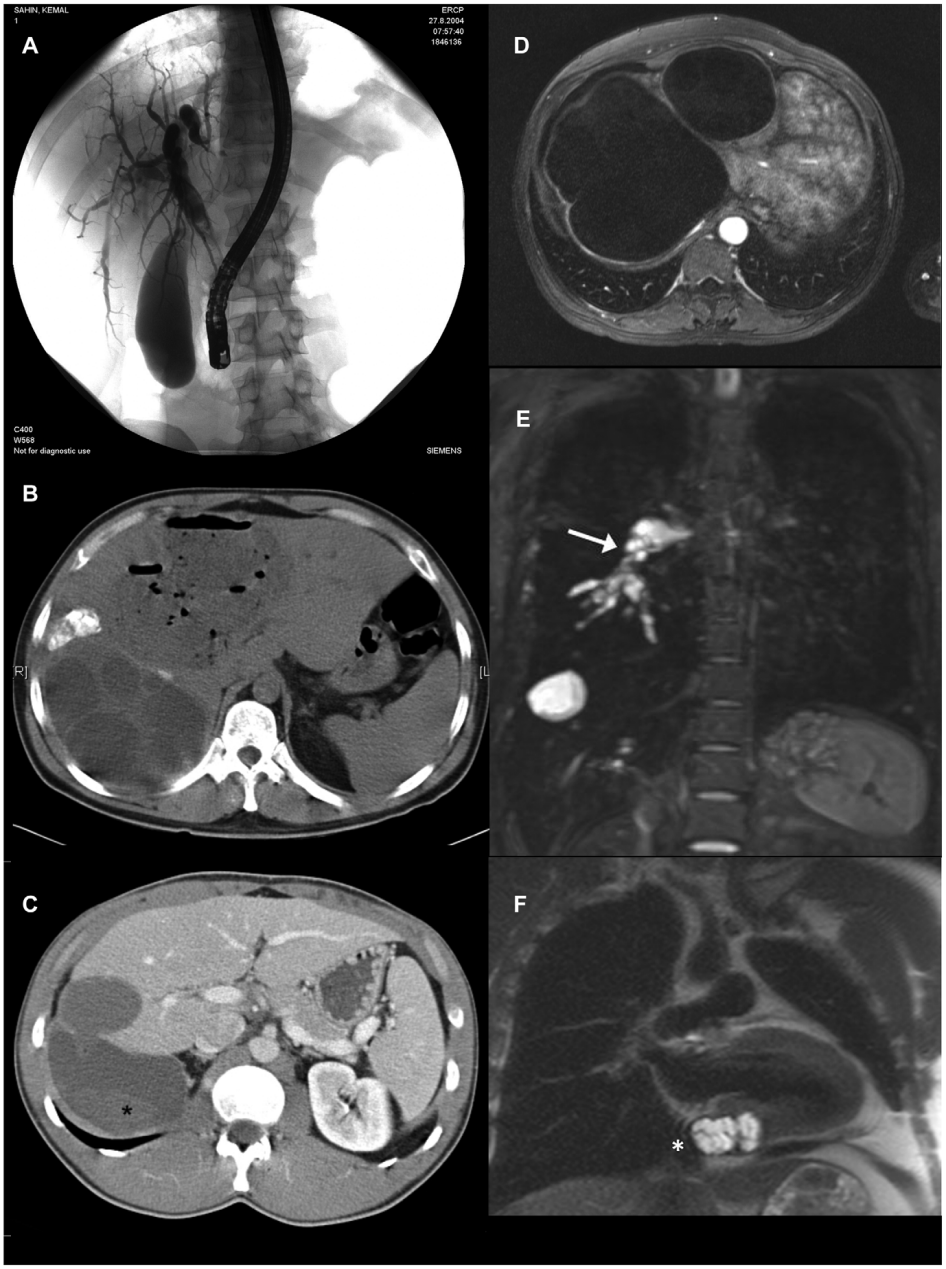
Severe and life threatening complications of CE. (A) Biliary obstruction/obstructive cholangitis due to biliary fistulas. (B) Liver abscess formation due to secondary bacterial infection of cysts. (C) Cyst rupture (*) followed by anaphylaxis and secondary echinococcosis. (D) Cysts exerting pressure on vital neighbouring structures (e.g., liver veins resulting in Budd-Chiari Syndrome). (E) Embolism of the right pulmonary artery (arrow) caused by cardiac CE and vascular invasion. (F) CE infestation of the posterior wall of the left heart replacing the myocardial layer at the base of the heart. (We thank W. Hosch, Department of Radiology, and A. Stiehl, Department of Gastroenterology, University Hospital Heidelberg, for the images.).

*(b) Patients developing complications.* Successful management depends on equipment, skills, and quality of available health services. The most common complications are biliary obstruction with or without cholangitis, bronchial obstruction, bacterial infection of the cyst cavity with abscess formation, rupture with anaphylactic reactions that range from mild to lethal anaphylactic shock, secondary echinococcosis (growth of new cysts caused by seeding of protoscolices, generally in a cavity such as the peritoneal space) following spillage of fluid from a cyst that ruptured either spontaneously or because of a therapeutic maneuver, and impaired function of organs and blood vessels compressed by growing adjacent cysts (Figure 2). In most endemic countries, the required setup is only met in major cities a long way off from where patients experiencing complications live.

## What Is Available Today to Diagnose and Treat CE Patients?

**Figure 3:**
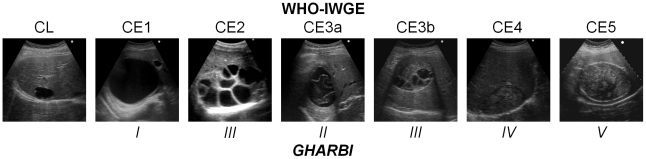
Comparison of Gharbi's and WHO-IWGE ultrasound classifications of CE cysts. CL, as a potentially parasitic cyst, was not in Gharbi's classification and needs to be differentiated from non-parasitic cysts. This may also happen with CE1 cysts, when the double layer sign is not evident. Also, WHO CE3b had not been explicitly described by Gharbi but could likely be classified as Type III.

Ultrasound is well established as a tool to diagnose, stage, and follow up CE cysts in the liver and other locations. Gharbi and colleagues developed the first widely adopted US classification in 1981 [10]. Other classifications were subsequently produced but were not as widely used. In 1994, the World Health Organization (WHO)-Informal Working Group started developing an international standardised US classification that could be universally applied to replace the plethora of classifications previously used (Figure 3) [11]. Even with all the obvious advantages of a standardised classification, some important issues still need to be resolved, one being the right sequence of cyst stages seen as the effect of natural or treatment-induced involution. A recent assessment of metabolic profiles of cyst stages with high-field proton magnetic resonance spectroscopy (^1^H MRS) has shown that the WHO IWGE classification of active, inactive, and transitional stages is perfectly in line with the metabolic activity profiles of the cysts, with the exception of CE3b, which appears vigorously active in ^1^H MRS, a finding that corresponds well with clinical experience [12]. US has been confirmed as an invaluable tool to assess cysts both with respect to viability and potential complications (Figure 2).

There are basically four management options: surgery, percutaneous sterilization techniques, anti-parasitic treatment, and observation (“watch & wait”). Their individual roles were recently reviewed [2]–[4]. Each of the four strategies certainly has its place, but the specific places and boundaries are still not well defined.

Surgery, the oldest form of treatment, keeps its place in most of the complicated forms of the disease. There is some competition between surgery and percutaneous approaches, in particular modified catheterization techniques, to be resolved, but this comparison requires carefully designed studies and cannot be decided on the basis of exclusively non-comparative small clinical studies, which are the only ones currently available.

Proponents of classical PAIR (punction, aspiration, injection, reaspiration) [13] have lost a bit of their enthusiasm after realizing that some cyst stages, such as CE2 and CE3b, are quite tedious to needle with too many compartments to be individually approached. But most importantly, these stages tend to relapse after PAIR. It remains to be seen whether large modified catheterization techniques can substitute for PAIR in these stages.

Over the past decade, several studies have been published suggesting that medical therapy (mebendazole, albendazole) could be an alternative to invasive treatment options in patients with uncomplicated cysts, broadening the indication for medical treatment over the years. The individual studies were all small and heterogeneity precluded appropriate meta-analysis. A recently published pooled analysis of individual patient data collected from six treatment centres suggests that the overall efficacy of benzimidazoles has been overrated [14]. Clinical trials stratified by cyst stage are needed to define the place of anti-parasitic treatment in the treatment of CE since it appears that it works better in some cyst stages (e.g., small CE1 cysts) than in others. The rate and nature of side effects of prolonged application of benzimidazole also deserves to be investigated more rigorously. Other anthelmintics, old and new (praziquantel, nitazoxanide), and combinations of anthelmintics (e.g., albendazole plus praziquantel) need to be properly investigated, too.

Though so far not systematically studied, experience with leaving certain cysts completely alone and only following them up over years, points to a fourth managing option, watch & wait. Apart from being biologically plausible, long term follow-up of patients with CE4 and CE5 cysts in anatomically silent corners of the body looks good. This holds great promise for patients in whom cysts have reached this stage and needs to be urgently systematically studied.

## Reasons for Arrested Progress in CE

Difficult, chronic diseases with a low case fatality rate clustering in poor rural areas are particularly “unattractive” to researchers and funders who depend on quick results to maintain the momentum of their activities. CE shares this fate with other communicable diseases, such as neurocysticercosis and Buruli disease. Health services also turn a blind eye on them since they plainly lack the means to manage patients with complex diseases such as CE appropriately. This is reflected in the low attention national and international institutions are paying to CE despite its substantial global burden, which is estimated at over 1 million DALYs per year [15], [16]. Additionally, due to its global distribution pattern, CE is not taking advantage of the attention that is being paid to “tropical” diseases. Interestingly, CE never made it to the list of the “TDR diseases” (from the WHO Special Programme for Research and Training in Tropical Diseases). The scarcity of resources and lack of momentum leads research to develop in niches with research communities too small to plan and conduct projects on a scale that allows conclusive answering of the relevant questions on efficacy, effectiveness, adverse reactions, and costs of a given treatment in comparison to other options. Currently available data arise from a multitude of small underpowered studies carried out over years, leading to contradicting results and recommendations, and, consequently, to controversies and difficulties (e.g., randomization) when planning appropriately designed clinical trials.

## What Do We Need to Improve CE Management in the Short Term?

Here is a most clinically neglected parasitic disease that urgently needs attention. A valuable tool for diagnosing, staging, and following up patients, ultrasound, is readily available. Four management procedures, surgery, percutaneous sterilization techniques, anti-parasitic treatment, and watch & wait, have “evolved” over decades, and been recently summarized [4], but without adequate comparative evaluation of efficacy, effectiveness, rate of adverse events, relapse rates, and cost. Clinical decision making is on even shakier ground for extrahepatic and extrapulmonary locations, which are rarer (see [4] for a list of extrahepatic and extrapulmonary locations with related treatments), and numbers needed to build comparative trials hard to come by. There is an obligation to put at least what we have on an appropriate evidence base by conducting comparative clinical trials at the scale and quality that allow answering these important questions. As one of the expected results, clear criteria for the watch & wait option alone might already save a substantial proportion of patients from unnecessary interventions and save health services money. Difficult chronic diseases clustering in poor rural areas need intelligent, creative approaches, and this one urgently needs operational research incorporating the particularities of resource-poor settings into consideration.
